# Incidental maternal glutaric aciduria type I detection through newborn screening: A case report

**DOI:** 10.1016/j.ymgmr.2026.101300

**Published:** 2026-02-26

**Authors:** Pierre-Edouard Grillet, Cecilia Marelli, Etienne Mondésert, Marie-Céline Francois-Heude, Agathe Roubertie, Frédérique Sabourdy, Jean-Paul Cristol, Cécile Acquaviva, Stéphanie Badiou

**Affiliations:** a Department of Biochemistry and Hormonology, Univ Montpellier, CHU Montpellier, France; b PhyMedExp INSERM, CNRS, Univ Montpellier, CHU Montpellier, France; c Reference Center for Neurogenetic Diseases, Univ Montpellier, EPHE, INSERM, CHU Montpellier, France; d LBPC-PPC, Univ Montpellier, INM INSERM, IRMB CHU Montpellier, France; e Department of Neuropediatrics, CHU Montpellier, France; f INM, INSERM U 1283, Montpellier, France; g Biochemistry Laboratory, CHU Toulouse, INSERM 1037, CNRS 5071, Université Toulouse III-Paul Sabatier, Centre de Recherches en Cancérologie de Toulouse (CRCT), Toulouse, France; h Department of Biochemistry and Molecular Biology, Inborn errors of metabolism unit, Hospices Civils de Lyon, Lyon, France; i Filière maladies héréditaires du métabolisme (G2m), Paris, France

**Keywords:** Newborn screening, Glutaric aciduria type I, Incidental diagnosis

## Abstract

The expansion of newborn screening in France (2023–2025) to include carnitine metabolism disorders has increased false positives, often due to unsuspected maternal metabolic conditions. We report the first french incidental diagnosis of a glutaric acidemia type I in a mother following a low C0 carnitine level detected on her newborn's screening. Genetic analysis revealed a previously undescribed mutation in the *GCDH* gene at a homozygote state consistent with an asymptomatic but high-excretor biochemical profile.

## 1 Introduction

Glutaric aciduria type 1 (GA-1) is a neurometabolic disease due to deficiency of glutaryl-CoA dehydrogenase, a mitochondrial enzyme responsible for glutaryl-CoA dehydrogenation affecting the catabolism pathway of lysine, hydroxylysine and tryptophan. The deficiency leads to accumulation of glutaric, 3-OH glutaric, glutaconic acids and glutarylcarnitine (C5DC-carnitine).

The disease is caused by an autosomal recessive bi-allelic pathogenic variants in *GCDH* gene located on chromosome 19p13.13. To date more than 290 *GCDH* different variants have been associated with GA-1 [1], [2], with different levels of enzymatic residual activity and therefore, biological phenotype separating two groups: low excretors (3–30% residual activity) and high excretors (0–2% residual activity), without correlation to the clinical presentation, as low excretors biochemical phenotype can lead to severe clinical presentations. Estimated global prevalence is about 1 / 100,000 newborns and some communities appears to have a higher frequency such as Amish people (Pennsylvania, USA), Oji-Cree First Nations (Canada), Irish travelers, Lumbee (North Carolina, USA) and Xhosa (South Africa) with up 1 / 300–400 newborns affected by GA-1 [2], [3], [4].

Most patients are asymptomatic during the first weeks of life or solely develop nonspecific neurologic symptoms (hypotonia or delayed motor development). Congenital macrocephaly is usually noticed at birth and precedes the severe neurological disease. In the classical acute-onset form, infants will present acute brain injury around the 9th month (3-36th month), usually trigged by an infection (gastrointestinal or respiratory upper tract) or during long fast, surgery or routine immunization. These encephalopathic crises will cause acute striatal damage responsible for loss of neurological functions, severe hypotonia, permanent dystonia and, in some cases, to limited life expectancy related to secondary complications. Usually after crises, patients slowly recover from their acute brain injury, but some extra-pyramidal symptoms may persist. An insidious-form lead to progressive striatal damage revealed with less severe dystonia before 6 years. Finally, a late-onset form is observed in patients with revelation during adolescence/adulthood through unspecific neurological symptoms such as transient gait, headache, tremors and some brain MRI abnormalities but without striatal damage [5].

We report here, one case of late diagnosis of GA-1 in an adult patient, diagnosed incidentally in context of extension of the newborn screening (NBS) program in France in 2023 including among others, carnitine uptake deficiency screening.

## 2 Case report

NBS of a female neonate is performed at three days of life after a normal pregnancy without any incident. The baby is delivered at 37 weeks and 2 days of amenorrhea after labor induction due to several late miscarriages (2012 and 2018). The baby birth weight is 2665 g, she measures 45,5 cm and has a cranial perimeter of 34 cm (normal values: 32-36 cm).

Concerning familial history, the 35 years-old mother is born from of a consanguineous union and is not related to the father. The parents do not present notable signs of any pathology. There are two other children in the family without any signs of pathology, one boy born in 2010 (14 years old) and one girl in 2014 (11 years old).

During all the pregnancy, the mother avoided milk and meat but ate fish and eggs.

On the dried blood spot (DBS) sampled for NBS, first measurement of free carnitine (C0) was below the first decision cut-off of 7 μmol/L. According to the French national algorithm, all values of C0 ≤ 7 μmol/L need to be confirmed on the same DBS in duplicate (Table 1), adding measurement of the sum of some acylcarnitines (ACS: C3 + C5 + C5DC + C8 + C10 + C16OH) in the decision tree. In case of mean value of C0 retest between 4 and 6 μmol/L with ACS < 1 μmol/L a second DBS at 21 days is sampled. If C0 is confirmed to be ≤4 μmol/L newborn is highly suspect of carnitine uptake deficiency and is declared to the pediatric staff.

**Table 1:** Dried Blood Spot acylcarnitines assays of the newborn.

Acylcarnitines	Newborn DBS 1st test (μmol/L)	Newborn DBS mean of retest (μmol/L)
C0 – free carnitine	3,80	3,99
ACS	NA	1,03

In the present case, the mean C0 retest value was 3.99 μmol/L (4.02/3.96) therefore, the newborn was referred to pediatric neurology department. Recommendation for bio-clinical management of suspected CUD in France includes biological analysis in the newborn and its mother for acylcarnitine profile, plasma carnitine, urinary carnitine and a chromatography of urinary organic acids.

All explorations were normal in the newborn but confirmed a severe deficiency in carnitine for the mother, with a plasma total carnitine of 2.2 μmol/L and 1.8 μmol/L of free carnitine associated with urinary total carnitine within the reference range (14.2 μmol/mol creatinine) and undetectable urinary free carnitine. Interestingly, the plasma acylcarnitine profile of mother revealed, along with low level of C0, a marked increase in C5-DC-carnitine indicating a probable GA-1 (Table 2). Urinary organic acid profile of the mother shows strong increase in glutaric acid excretion (3972 μmol/mmol creatinine, control range < 10) associated with high 3-hydroxyglutaric acid, highly evocative of a GA-1 with classic biological phenotype. In addition, acylcarnitine profile in urine revealed a large amount of glutaric acid bound to carnitine (Table 2) in accordance with a high excretor GA-1 profile.

**Table 2:** Plasmatic and urinary acylcarnitine assays of the newborn and her mother. Reference values are specified in italic.

Acyl-carnitines	Newborn confirmation test on plasma (μmol/L)	Mother plasma (μmol/L)	Mother urine (μmol/mmol creat)	Mother plasma D + 12 day(μmol/L)
C0 – free carnitine	11,5 *(10–60)*	33,06 *(10–60)*	31,2 *(0,35–38)*	10,359 *(10–60)*
C2 – acetyl carnitine	3,8 *(3,5–30)*	0,590 *(3,5–25)*	0,094 *(<15)*	2,349 *(3,5–25)*
C3 – propionyl carnitine	0,594 *(<1,15)*	0,091 *(<1)*	0,022 *(<1,8)*	0,138 *(<1)*
C5 – isovaleryl carnitine	0,088 *(<0,5)*	0,014 *(<0,35)*	0,016 *(<1,8)*	0,032 *(<0,35)*
C5DC – glutaryl carnitine	0,036 *(<0,3)*	**2,355** *(<0,3)*	**31,967** *(<1,5)*	**6,407** *(<0,3)*
C8 – octanoyl carnitine	0,024 *(<0,25)*	0,007 *(<0,25)*	0,004 *(<0,9)*	0,054 *(<0,25)*
C10 – decanoyl carnitine	0,027 *(<0,35)*	0,008 *(<0,35)*	0,013 *(<0,4)*	0,084 *(<0,35)*
C14 – tetradecanoyl carnitine	0,011 *(<0,22)*	0,003 *(<0,05)*	0,001 *(<0,35)*	0,010 *(<0,05)*
C16 – hexadecanoyl carnitine	0,057 *(<0,55)*	0,022 *(<0,3)*	0,001 *(〈0,1)*	0,047 *(<0,3)*
C16-OH – hydroxyhexadecanoyl carnitine	0,001 *(<0,03)*	0,001 *(<0,01)*	0,001 *(<0,05)*	0,001 *(<0,01)*

The suspicion of GA-1 in the mother motivated a new consultation in the metabolic department for further explorations. The anamnesis reports no specific sign of abnormality during the psychomotor development, only eyebrow dyskinesia is observed. She has a college education level.

Her cranial perimeter is normal (58 cm) consistent with the absence of macrocrania.

A molecular study was performed for the mother revealing an homozygous pathogenic variant in *GCDH* (NM_000159): c.641C > T (p.Thr214Met). The variant was previously reported in literature in a series of Irish patients [6] and two case reports [7], [8] of GA-1 patients.

**Fig. 1:**
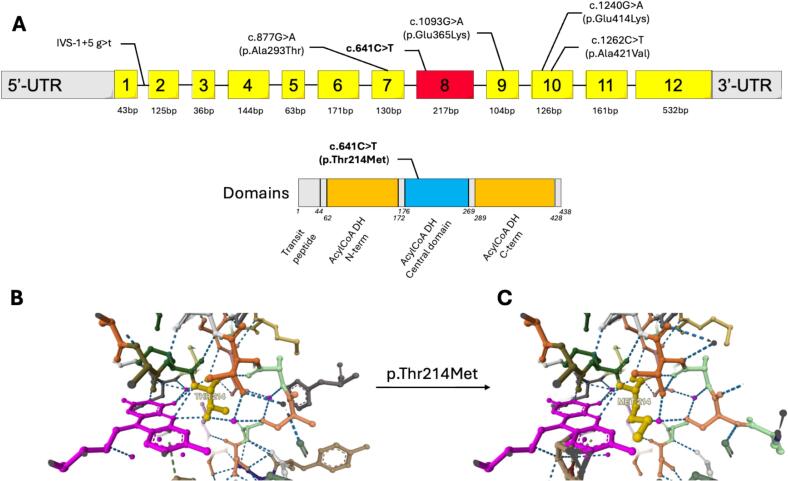
A. GCDH variants and Glutaryl-CoA dehydrogenase domains localization of most frequent variants and the one of interest. The variant of interest is depicted in bold, the others described in some communities such as Amish people (Pennsylvania, USA, c.1262C>T), Oji-Cree First Nations (Canada, IVS-1+5 g>t), Irish travelers (c.1093G>A), Lumbee (North Carolina, USA, c.1240G>A) and Xhosa (South Africa, C.877G>A) are figurated with their position on the GCDH gene (NM_000159.4). Three dimensional modeling structure analysis of the c.641C>T (p.Thr214Me)t mutation on the ligand FAD interaction binding site. Panel B shows the three-dimensional structure of the wild-type protein depicting 3 hydrogen bounds between the 214 residue threonine and FAD whereas panel C, presents the mutated protein, with a 214 residue methionine showing only 2 hydrogen bounds with FAD, wich may affect the enzyme function by inhibiting its ligand binding.

Interestingly, to date, this variant has never been reported at the homozygous state. The analysis of this variant using MobiDetails [9] tool showed that it is not a member of the most common known mutants such as c.1204C > T (Caucasian), c.1262C > T(Pennsylvania Amish community), c.91 + 5G > T (Oji-Cree first nations Canadian community) or c.1093G > A (Irish community travelers) (Fig. 1A). Clinical interpretation of the variant with ClinVar presents the variant as Pathogenic/Likely pathogenic, as InterVar [10] which classify it, as likely pathogenic (PM1, PM2, PM3, PP3, PP5).

The mutation replaces a threonine by a methionine involving a change from a polar to a non-polar residue in position 214 at the beginning of the 8th exon (6 bp from the acceptor) (Fig. 1A).

As suggested by 3D structure protein prediction, the mutation is responsible of a lost interaction between the enzyme and the co-factor flavin adenine dinucleotide (FAD) due to a steric clash, that could explain the enzyme loss of function.

During the metabolic consultation, a prescription of oral levocarnitine was proposed but was not followed by the patient due to poor tolerance. A dietary consultation revealed a natural aversion for meat and a self-made low protein diet with 0,6 mg/kg/day.

Unfortunately, the patient refused to achieve a complete evaluation of her disease nor a follow-up and canceled the other examination, notably the brain MRI.

## 3 Discussion

Since January 2023, the French NBS program implemented seven new diseases to its panel (CUD, GA-1, Long-Chain 3-Hydroxyacyl-CoA Dehydrogenase deficiency, Tyrosinemia type I, Isovaleric aciduria, Homocystinuria and Maple Syrup Urine Disease) and VLCAD (Very Long Chain Acyl-CoA Dehydrogenase deficiency) in September 2025, to improve the diagnosis and early treatment for these rare but severe and treatable diseases. Implementation of a rare disease in NBS is likely to increase its prevalence in the following years and also discovering maternal cases and new phenotypes/genotypes correlations. The present case report is the demonstration of this aspect and highlights the need for clinicians and medical biologists to apply strictly algorithms extending the explorations to the mother for suspected newborn of CUD or GA-1.

• In the present case, the NBS leads to suspicion of CUD, but all analyses of the confirmation step were normal for the newborn. By contrast analyses performed in the mother are compatible with GA-1 diagnosis, never suspected to date.

The false positive result of CUD in the newborn was related to the low level of free carnitine available from the mother to her child during pregnancy, explained by the massive esterification of glutaryl-CoA to carnitine in the mother. The observation of false positive in NBS of CUD due to maternal causes was previously reported [8], [11], [12], [13], [14] but, this is the first occurrence in France. Of note normal C5DC-carnitine value was observed on the NBS (0,15 μmol/L / suspect cut-off: 0,5).

The second originality of this case report, is the first description report of c.641C > T (p.Thr214Met) variant in homozygous state*. In silico* modelisation of the structural consequences of this variant reveal a potential steric clash inhibiting the association between the enzyme and its cofactor FAD (Fig. 1B–C). Although we didn't study the functional activity of the mutated enzyme, we could speculate that the residual activity is likely below <2% due to the high excretor profile of the patient [15]. The clinical phenotype appeared to be mild since the medical examination revealed no particular sign of brain injury and no reports of encephalopathies during the infancy by the patient, highlighting the lack of relation between biological phenotype and clinical outcome. Unfortunately, no brain MRI was performed nor other clinical or laboratory examinations due to the refusal of patient. Other reports will be needed to correlate or not, this mutation to an asymptomatic/mild symptomatic phenotype. National and European/Global newborn screening program will be helpful to bring up other reports of this mutation or new mutations that will be of interest to better understand and characterize GA-1 especially for incidental detection of late onset/asymptomatic forms. These programs will also help physicians and scientific societies in producing clinical recommendations to treat and follow these adult patients suffering from diseases usually detected in infancy.

## CRediT authorship contribution statement

**Pierre-Edouard Grillet:** Investigation, Writing – original draft, Writing – review & editing. **Cecilia Marelli:** Writing – review & editing. **Etienne Mondésert:** Writing – review & editing. **Marie-Céline Francois-Heude:** Writing – review & editing. **Agathe Roubertie:** Writing – review & editing. **Frédérique Sabourdy:** Writing – review & editing. **Jean-Paul Cristol:** Writing – review & editing. **Cécile Acquaviva:** Writing – review & editing. **Stéphanie Badiou:** Investigation, Supervision, Validation, Writing – review & editing.

## Declaration of competing interest

The authors declare that they have no known competing financial interests or personal relationships that could have appeared to influence the work reported in this paper.

## Data Availability

Data will be made available on request.
